# Geometrical and Mechanical Properties Control Actin Filament Organization

**DOI:** 10.1371/journal.pcbi.1004245

**Published:** 2015-05-27

**Authors:** Gaëlle Letort, Antonio Z. Politi, Hajer Ennomani, Manuel Théry, Francois Nedelec, Laurent Blanchoin

**Affiliations:** 1 Laboratoire de Physiologie Cellulaire et Végétale, Institut de Recherches en Technologies et Sciences pour le Vivant, iRTSV, CNRS/CEA/UGA, Grenoble, France; 2 Laboratoire d'Imagerie et Systèmes d'Acquisition, CEA, LETI, MINATEC Campus, Grenoble, France, Univ. Grenoble-Alpes, Grenoble, France; 3 Cell Biology and Biophysics Unit, EMBL, Heidelberg, Germany; Weizmann Institute of Science, ISRAEL

## Abstract

The different actin structures governing eukaryotic cell shape and movement are not only determined by the properties of the actin filaments and associated proteins, but also by geometrical constraints. We recently demonstrated that limiting nucleation to specific regions was sufficient to obtain actin networks with different organization. To further investigate how spatially constrained actin nucleation determines the emergent actin organization, we performed detailed simulations of the actin filament system using Cytosim. We first calibrated the steric interaction between filaments, by matching, in simulations and experiments, the bundled actin organization observed with a rectangular bar of nucleating factor. We then studied the overall organization of actin filaments generated by more complex pattern geometries used experimentally. We found that the fraction of parallel versus antiparallel bundles is determined by the mechanical properties of actin filament or bundles and the efficiency of nucleation. Thus nucleation geometry, actin filaments local interactions, bundle rigidity, and nucleation efficiency are the key parameters controlling the emergent actin architecture. We finally simulated more complex nucleation patterns and performed the corresponding experiments to confirm the predictive capabilities of the model.

## Author Summary

Many cellular processes or morphological changes, for example division and migration, strongly depend on the cytoskeleton. For successful completion of these processes in higher cells, thousands of cytoskeletal filaments need to be organized into precise higher-order structures. Identifying the most important parameters governing the emergent organization of large systems of filaments is thus a crucial step in understanding cytoskeletal systems. However, due to the complexity of these systems and the limits of imaging, this has proven to be a difficult task. To overcome this limitation we previously established an *in vitro* assay for actin filament assembly that allows controlling both the chemical components and geometrical boundaries. Here we used detailed computer simulations to rationalize the experimental observations and identify some of the physical principles governing actin organization. Such approach will ultimately allow one to predict actin network formation under various constraints.

## Introduction

Actin assembles to form higher order structures [1] that are essential to cell morphogenesis, adhesion and motility [2]. A single filament can either resist or generate forces according to its local environment [3,4], but most physiological processes require the assembly of a higher ordered network [5,6]. Therefore, one needs to study the collective behavior of system composed of many thousands of actin filaments to understand their physiological functions. Indeed, whereas one actin filament could not sustain forces higher than a few pN [4], a bundle of actin filaments can resist hundreds of pN [7] and larger structures are able to bear even higher forces (nN range) [8]. In addition to the number of filaments, the architecture of the network is also adapted to achieve different cellular functions. At the edge of the cell, in the lamellipodium, actin filaments form a dense branched network [9–11], that seems optimal to push the membrane forward during actin-based motility [12,13]. Actin filaments are oriented with their elongating ends near the membrane, at an optimum angle of ± 35° with respect to the membrane [14, 15, 16], and being present in high density close to the membrane [17], they can efficiently sustain the protrusive force [13]. Actin filaments can also form bundles of parallel filaments creating finger-like protrusions in the membrane called filopodia [18] that explore the extracellular matrix [19]. Actin bundles can be also used as tracks for protein or cargo transport [20,21]. These bundles can be formed by branched organization that merged into elongated parallel actin filament [22,23]. Finally, actin filaments can form aligned structures of anti-parallel filaments in stress fibers or transverse arcs, that are site of active contraction driven by myosin motors [24,25] and are responsible for the cellular mechanical response [26]. Overall, the architecture of an actin network is expected to be directly related to its physiological function. The binding partners guide the organization of actin filaments, and conversely the binding of actin-associated proteins is sensitive to the architecture of the actin network [27,28]. Deciphering the principles governing the assembly of the different actin structural architectures is an important step towards a better understanding of the variety of cellular processes. Many excellent studies have focused on identifying the biochemical composition of the different actin organizations [6], but the physical and geometrical laws governing their architecture are still largely unknown.

In a previous work, we developed an *in vitro* method to control actin filaments assembly with a designed pattern composed of areas with different surface properties [1]. In these experiments, the geometry of the nucleation zone dictated the collective behavior of the actin filaments with some patterns resembling *in vivo* like structures [1]. The system was built from a minimal set of purified proteins, and avoiding the biochemical complexity of an *in vivo* system, it provided a well-controlled and reproducible assay to study the assembly of actin filaments in a variety of structures. Although, our study revealed how geometrical constraints affect actin assembly, the key components responsible for the formation of defined structural organizations remained poorly defined. The stochastic model developed in this study [1] helped to describe the observation but did not bring additional information on the properties of the system dictating the collective behavior. To better identify these parameters and ultimately understand the higher order architecture, we need to be able to predict the emergent actin organization, based on the microscopic properties such as the rates of actin assembly, the mechanical properties of actin filaments, the geometry of the nucleation region, and the biochemical composition of the experimental system.

Different types of modeling are available for this purpose [29]. Collective cytoskeleton behavior has been studied at macroscopic scale with ordinary or partial differential equations [30–32]. Stochastic methods (Monte Carlo simulations) can take into account the variability arising from the intrinsic randomness of the microscopic processes [15,33–35]. The potential drawback of such detailed modeling is that they can be computationally expensive, but with modern methods [29], one can simulate systems containing thousands of filaments over hundreds of seconds. We used Cytosim [36], a versatile cytoskeleton simulation software, which can be used for a diverse range of cytoskeleton simulations. Actin filaments can be simulated with different growth and shrinkage rates and bending elasticities. Associated proteins can be added (e.g. molecular motors, crosslinkers, severing proteins, capping proteins, nucleators), and environmental constraints can be imposed (confinement, asters, solid objects, laser cutting, flow…). Cytosim polymer has been used to study microubule systems: self-organization [37,38], effect of confinement [39], spindles [40], asters positioning [41], nuclear positioning [42] but also to predict experimental design [43]. Nonetheless, Cytosim had never been used to study actin assembly under geometrical constraints.

Numerical experiments based on similar modeling have already been used to study the parameters controlling the global organization of cytoskeleton components. Recently, it was shown how different modes and efficiency of actin filament crosslinking would affect the self-organization of actin structures such as the contractile ring and actin cables [44, 45]. Similarly, simulations of taxane-stabilized microtubules [46] showed that geometrical constraints (cell confinement) combined with the bending properties of the filament was sufficient to create the bundling effect observed *in vitro*.

In this work, we first designed a simulation of actin organization from a nucleation region with a defined geometry, and reproduced the *in vitro* collective actin assembly behavior, with a minimum set of parameters. We then used this system to investigate how these parameters control the network organization in a range of experimental conditions. This study showed that we have identified the key parameters that define geometrically-controlled actin assembly and are able to reproduce a variety of actin organization *in silico*. Thus we believe that we have in hand a powerful predicting tool, and that numerical simulations in combination with *in vitro* experiments will help understand more complex actin processes.

http://www.ncbi.nlm.nih.gov/pubmed/23388829

## Results

**Fig 1:**
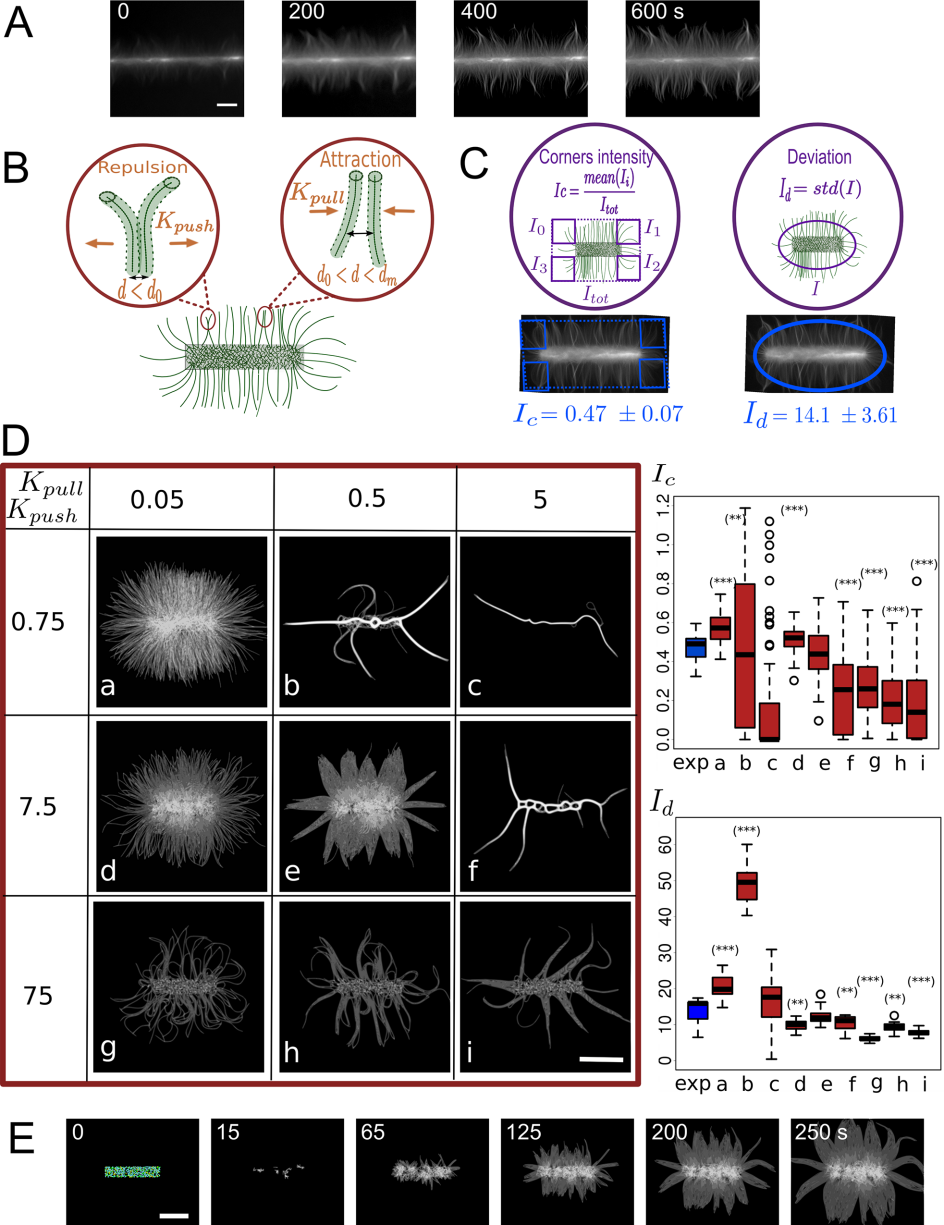
Parameter calibration for actin growth on patterns. (A) *In vitro* actin filaments growing from an horizontal bar coated with the nucleation promoting factor pWA. Scale bar is 10 μm. (B) Schematic of steric interactions: the filaments repulse each other at short distance (*d*<*d*_0_), and attract each other at medium range (*d*_0_<*d*<*d*_*m*_), and do not interact at longer range (*d*>*d*_*m*_). Attractive and repulsive interactions are characterized by different stiffness (*K*_*pull*_, *K*_*push*_) that are applied to points distributed every 0.2 μm along the filaments. (C) To compare simulated and experimental data we used (left panel) the ratio *I*_*c*_ of average intensities in areas adjacent to pattern corner (*I*_i_) by the total image intensity (*I*_*tot*_). Both values are normalized by area size. To compare the bundling behavior, we measured **I**_d_ the standard deviation of the intensity along an ellipse around the pattern (thus giving us the fluctuations of the distribution of filaments, right panel). Average corners intensity *I*_*c*_ and intensity deviation *I*_d_ from 10 experimental images (below images). (D) Variation of steric parameters and comparison of simulated and experimentally estimated *I*_*c*_ and *I*_d_. Left: Three values were tested for *K*_*pull*_ (0.05, 0.5 and 5 pN/μm) and three values for *K*_*push*_ (0.75, 7.5 and 75 pN/μm), resulting in 9 combinations. The nucleating bar is 8 μm long and each image shows grey levels of actin density from one simulation at 250 s (scale bar is 5 μm). Right: Intensity ratio *I*_*c*_ and intensity deviation *I*_d_ for each set of parameters (**a-i**, red, from 20 simulations each) and experimentally estimated ratio (exp, blue). Based on their intensity ratios, the simulations shown on **d** and **e** agree well with experiment, while the ones shown on **b**, **c**, **f**, **g**, **h**, **i** are significantly different from experimental results (p-value < 0.001 (***), 0.005 (**), 0.01 (*), Kolmogorrov-Smirnov test). With the same test, conditions **a**, **b**, **d**, **f**, **g**, **h**, **i** are significantly different from experimental results based on intensity deviation analysis. (E) Simulated growth of filaments using set **e** (*K*_*pull*_ = 0.5 pN/μm, *K*_*push*_ = 7.5 pN/μm). Actin filament nucleation is triggered by contact between primers with Arp2/3-like objects placed in the central rectangle (see S1 Fig). Scale bar is 5μm. The pattern is 8 μm wide and time in seconds is indicated.

To study geometrically-controlled actin assembly, we previously developed an assay where actin nucleation is triggered from a micropatterned surface coated with an Nucleation Promoting Factor (NPF) (Fig 1A and [1]). The assay includes crowding agents constraining the filaments parallel to the glass. In this assay, actin nucleation occurs near the glass surface, and the growing actin structures never exceed 7μm in height, as revealed by confocal imaging (S1 Fig). The highest rise occurs immediately above the nucleating regions, while most of the network that extends away from them seems to be within 200 nm of the glass surface. This is confirmed by the fact that confocal and TIRF images of the system are comparable. The 2D model (S2 Fig) developed in Cytosim seems therefore appropriate to simulate the layer of actin assembly that is directly in contact with the glass, which is the part of the network that is most interesting in our experimental condition.

To simulate a geometrically constrained actin nucleation as used in experiments [1] we randomly disposed non-diffusible Arp2/3-like entities within the desired area (see S2, S3 Figs and Material and Methods). These Arp2/3 complexes are able to nucleate a new (daughter) filament, but only if they are already bound to an existing (mother) filament. Because they do not move by diffusion, and thus remain in the nucleating region, the Arp2/3 complexes can generate branches within the nucleation area, but not outside. This mimics the activation of the Arp2/3 complex by the pWA-peptide coated on the pattern (S2 Fig). The Arp2/3 complex acts as a mechanical link between mother and daughter filaments, constraining them relative to each other in position and in orientation, such that the pointed end of the daughter filament remains on the side of the mother filament, and the daughter barbed end grows out making a ~70° angle with the mother filament [47, 48]. In the same area, we also added fixed nucleators, which initiate the process by generating the primer filaments, and fixed binders, which may bind to any actin filament within their range, and are anchored at a fixed position with a Hookean spring. These “fixed binders” have a non-zero unbinding rate and thus they effectively create some friction acting on any filament entering the micro patterned region. This friction accounts for the constrained nucleation processes and is necessary to maintain a dense patterned area (S2 Fig).

The simulated filaments are not meant to only represent individual actin filaments, but may also represent bundles of several crosslinked filaments. We will thus refer to them as fibers. Similarly, the ‘steric’ interaction between fibers is meant to be effective, representing the different forces that may act between neighboring F-actins. These parameters can be varied experimentally by modifying the type and concentration of actin binding proteins, the buffer solution or fiber confinement, conditions that have been shown to induce different actin organizations [1]. Our first task was to calibrate the effective parameters of the simulation, by comparing simulations and experiments obtained with a simple pattern.

### Role of actin filament steric interactions in determining the actin organization

Input of Cytosim is a configuration file specifying the values of all parameters of the model: some represent physical quantities (e.g. temperature, viscosity), some are associated with the algorithm of the simulation (e.g. time step, segmentation) and were set to get an appropriate precision. Yet, most parameters are characteristics of the real components of the system (e.g. F-actin growing speed and bending rigidity). We adopted measured values when possible, using for example the measured persistence length of 15 μm of actin filaments [6] and a rate of elongation of 0.033 μm/s (actin concentration of 1–2 μM [49]). Finally, a few parameters are associated with effective interactions for which a molecular implementation is not necessarily feasible. This is the case in particular for filament-filament interactions, which are essential to capture the collective behavior of growing filaments [50,51]. Actin filaments are physically not able to interpenetrate each other and within distances equal to their diameter must experience strong repulsive force. Moreover, although similarly charged, actin filaments attract each other at short range, due to the presence of counterions in the solution [50,51]. Moreover, the presence of chemically neutral polymers in the solution creates a depletion effect that also induces neighboring filaments to pack together [51, 52]. This explains how we could observe bundles *in vitro* without adding any actin filament crosslinkers to the solution. It also means that changing the experimental conditions (pH of buffer, ionic strength, concentration of polymers, etc.) may affect how actin filaments interact. Rather than trying to simulate these processes in details, we have used an ad-hoc steric interaction between simulated filaments, and calibrated the parameters of this interaction to best reproduce the behavior of the *in vitro* system. In Cytosim, a ‘steric’ filament-filament interaction can include both attractive and repulsive forces (Figs 1B and S2):

$$F_{s}=k\;\left(\;d-d_{0}\;\right),\;with\;k=\;\{\begin{array}{c} K_{push}\;if\;d<\;d_{0}\\ K_{pull}\;if\;d_{0}\;\leq d\;\leq \;d_{m}\\ 0\;otherwise \end{array}$$ (1)

where *d* is the distance between the two interacting elements, *d*_*0*_ is the distance at which the fibers are at equilibrium (this is an effective fiber diameter, which is larger than the real diameter), *d*_*m*_ is the maximal interaction distance between fibers (the choice of these values is discussed in Material and Methods). To fix the two stiffness values, *K*_*push*_ and *K*_*pull*_ (Fig 1B), we tested a range of values and compared systematically the simulation outputs with experimental results at first using a simple pattern configuration: a horizontal bar (Fig 1A, 1C and 1D). On this pattern actin filaments assembled into a dense network on the patterned bar, with their barbed ends growing away from the pattern, filaments align parallel to each other and generate bundles growing out perpendicularly to the bar (Fig 1A and [1]). In the simulations we found that a balance between repulsing and attracting steric interactions strongly affects the organization of actin (Fig 1D). When the attractiveness was similar or higher than repulsion (Fig 1D panel b, c, f) actin fibers collapsed together. On the contrary, when the repulsion dominates (*K*_*push*_ = 75 pN/μm, Fig 1D, panel g, h, i), actin fibers are disorganized and do not generate bundle-like structure. For a quantitative comparison of simulations and experiments we computed the average local intensity in the corners of the patterned bar normalized by the total average intensity (*I*_*c*_, Fig 1C). This quantity gives a measure of the spatial distribution of the filaments: when organized in bundle-like structures, fibers are less dense in the corners of the pattern thus *I*_*c*_ is lower than 1. A bar plot of *I*_*c*_ in experiment (*N* = 10) and for the 9 simulation scenarios (*N* = 20) is shown in Fig 1D. We found that the corner intensities corresponding to panels a, b, c, f, g, h, i (Fig 1D) were significantly different from experimental ones (*p*-value < 0.005, Kolomogorov-Smirnov test), whereas the ones corresponding to panels d and e were not (*p*-value>0.1). To further compare simulations and experiments, we also measured the variation of intensity along the perimeter of an ellipse around the pattern (*I*_d_). This gives a measure of the bundling of the filaments: a high value indicates the presence of intense bundles, whereas a low value accounts for spread filaments all over the pattern. With this measurement, we found that the deviation of intensity corresponding to panels a, b, d, f, g, h, i were significantly different from experimental ones. Thus, panel e (K_push_ = 7.5 pN/μm, K_pull_ = 0.5 pN/μm) quantitatively reproduced the *in vitro* actin filaments behavior most accurately. Indeed, the actin network growing from the micro-pattern in simulation (Fig 1E and S1 Movie) was fully consistent with our experimental observations [1]. All following simulations thus used these calibrated parameters.

### Geometrical conditions and mechanical properties of filaments both impact on their structural organization

**Fig 2:**
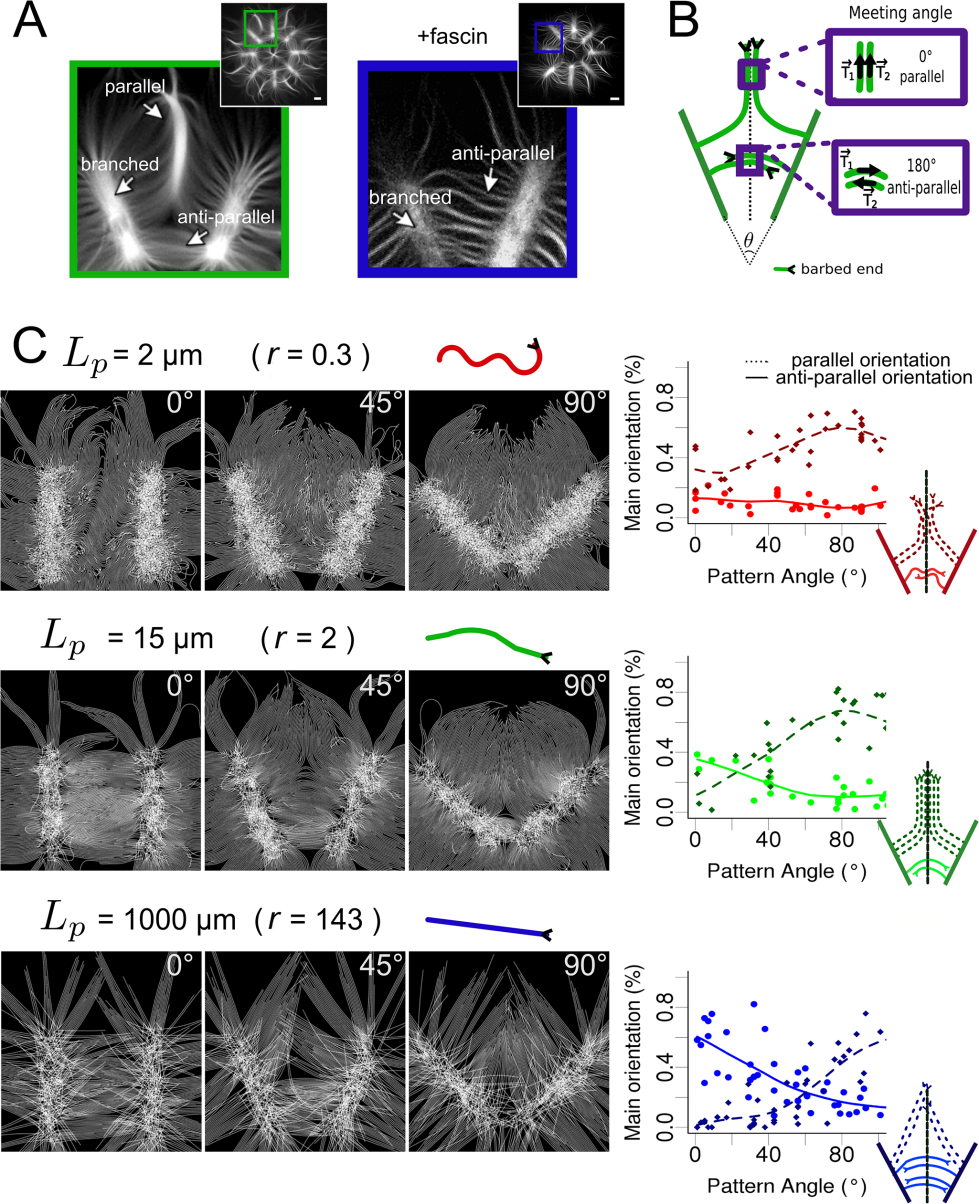
Effect of geometrical constraints and fiber mechanical properties on fiber organization. (A) *in vitro* actin filament organization from a star eight-fold radial array pattern coated with pWA activator, in absence (green) or presence (blue) of fascin, an actin filament cross-linker. A portion of the pattern containing the V-shaped submotif is shown at higher magnification in both cases. (B) Schematic representation of the actin organization on two adjacent rays (V-shaped configuration): parallel (top) and anti-parallel filament structures (bottom). The presence of these two patterns is assessed from the meeting angle between filaments from opposing patterns on the middle line. (C) Left: Simulated filament growth on patterns with 3 different angles (0°, 45°, 90°) and 3 different filament persistence lengths (*L*_*p*_ = 2 μm, r = *L*_*p*_/*L* = 0.3, red; *L*_*p*_ = 15 μm, *r* = 2, green; *L*_*p*_ = 1000 μm, *r* = 143, blue) (one simulation is shown in each case). Right: Proportions of the main orientation (percentage of filaments crossing the median line with a particular orientation) of filaments (dashed lines: parallel filaments, full lines anti-parallel filaments) on the median line, as a function of the angle between the two patterns.

When more than one area of nucleation is present on the pattern, the distance between them, and their relative orientation have a major impact on the emergent organization [1]. Indeed, actin filaments coming from two different actin networks may change their initial direction when contacting each other. A typical example illustrating this behavior is the global organization of actin filaments initiated by V-shaped branches of an eight-fold radial array (Fig 2A). Growing actin filaments elongating outward from each ray formed parallel bundles on the bisecting line between adjacent rays (Fig 2A, left panel). These parallel bundles originate from a transition point that separates the assembly of antiparallel bundles in the proximal part of the rays and the assembly of parallel bundles in their distal part (Fig 2A and cartoon Fig 2B). Depending on the angle *θ* between two patterns creating a V-shape motif (Fig 2B), actin filaments contact each other differently [1]. We also noticed that addition of a crosslinker (fascin) from the beginning of the experiment changed the final organization of the filaments thereby increasing the proportion of anti-parallel structures. A likely explanation for this is that crosslinked actin filaments generated by fascin behave differently than isolated actin filament (Fig 2A, right panel). One property affected by crosslinking is the stiffness of the resulting bundle. We therefore simulated V-shape patterns with different angles and varied the persistence length *Lp* of the simulated filaments, a quantity proportional to the bending stiffness *K* (*K* = *k*_*B*_ *T Lp*) (the results of varying the bending rigidity are further discussed in Material and Methods).

For a native persistence length of *L*_*p*_ = 15 μm (Fig 2C, middle panels and S2 Movie), fibers meeting with a shallow angle usually grow past each other and form anti-parallel structures (*θ* = 0°, meeting angles > 160°). On the contrary, fibers growing from two bars disposed at right angle (*θ* = 90°) tend to bend as they meet, resulting in parallel structures (meeting angles < 20°). For an intermediate angle (*θ* = 45°), the two types of arrangement coexist: as in experiment (Fig 2A) antiparallel structures are found in the bottom part between the two bars, and parallel structures in the top part. We found that the rigidity of the fibers (relative to the filament length) controls the proportion of parallel and anti-parallel structures. At high persistence length (*L*_*p*_ = 1000 μm, Fig 2C bottom panels and S4 Movie) corresponding to bundles of 8–10 F-actins [53], anti-parallel structures are more prominent, compared to the structures simulated with a native persistence length *L*_*p*_ = 15 μm. This behavior is reminiscent of the effect of fascin (Fig 2A, right panel). At small persistence length (*L*_*p*_ = 2 μm), corresponding to actin filaments decorated by ADF/cofilin [54], the distance between the bars is much greater than the persistence length and thus the fiber brushes deform upon interaction (Fig 2C top panels and S3 Movie).

We next analyzed the local orientation of actin fibers along the bisecting lines when we varied both the angles between the two bars and the polymer persistence length. We classified fibers whose orientation compared to the *x*-axis was around 90° ± 20° as parallel filaments, and those whose orientation was of 0° ± 20° as anti-parallel. The graphs (Fig 2C, right panel) show the proportion of different actin fibers orientations as function of the angle *θ* between the patterns (for an angle varied from 0° to 120°) for the 3 different persistence lengths. This analysis demonstrated the continuous dependence of the structures on the pattern angle.

These results can be rationalized as follows: since filaments initially grow primarily perpendicular to the bar, they need to bend by an angle *θ* /2 to enter an antiparallel structure, and by 90°—*θ* /2 to enter a parallel structure. For *θ* < 90°, the bending angle required to form an antiparallel structure is smaller than the one required for a parallel structure, which makes it unfavorable for stiff filaments to form a parallel structure, all the more if they are short. This is because given a certain force *F* applied transversally at the growing tip of the filament, its bending angle will scale as *θ* ***~*** *FL* ^***2***^ ***/*** *K*, where *K* is the filament bending stiffness (*K* = *k*_*B*_ *T Lp*) and *L* the length of the actin filament. At the bottom of the pattern, actin filaments meet while they are short, the bending required to enter a parallel structure is consequently unfavorable, and anti-parallel structures are created instead. On the top of the pattern however, actin filaments are longer as they contact opposite filaments; and consequently are easier to bend leading to parallel bundles (Fig 2B and 2C).

To further confirm this analysis, we repeated these simulations with fibers of 15 μm persistence length and varied the length of the fibers (and thus the distance between the patterns and the simulated time as well). The analysis of the presence of structures (S4 Fig) revealed that indeed the ratio between the polymer persistence length and their length will determine their collective behavior. Indeed, short actin fibers with a persistence length of 15 μm will have the same behavior than shorter fibers with 2 μm persistence length or longer fibers with 1000 μm persistence length (S4 Fig). The definition of the persistence length makes this relation between filament length and rigidity obvious for isolated filaments, but was necessary to demonstrate in the context of collective filaments behavior.

Thus, both the geometrical conditions of nucleation and the mechanical properties of the simulated filaments are key parameters controlling the formation of anti-parallel or parallel structures.

### Actin filaments reaching a branched network can cross it and/or generate new filaments by nucleation

**Fig 3:**
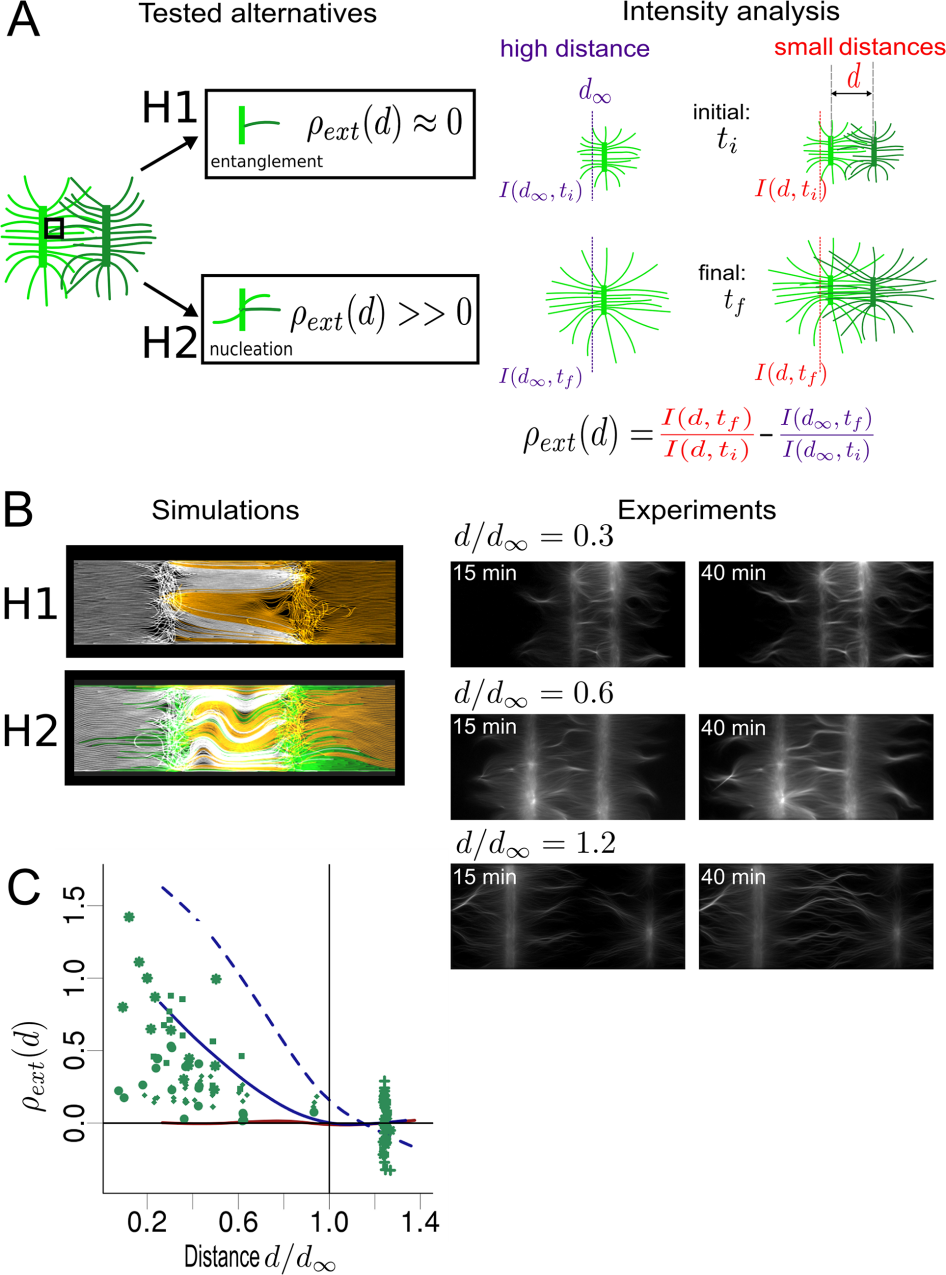
Actin fibers growing from adjacent patterns influence the overall actin organization. (A) Assumptions tested and analysis performed. Left: Two different hypotheses concerning the activity of a filament reaching a nucleated pattern were tested: H1 (“entanglement”) and H2 (“nucleation”). Under H1, filaments stop growing and do not induce new nucleation. Under H2, filaments induce the nucleation of new filaments on the pattern that they reach. To determine which hypothesis is valid, we measured the variation of intensity in an area close to the pattern (in the outer side), and compare this variation with a control case corresponding to an isolated pattern (in the experiments, patterns separated by a distance *d* > = 80 μm are considered as isolated; in the simulation the threshold distance is *d*_*∞*_ = 10 μm) (right). This variation (*ρ*_*ext*_) indicates the amount of new filaments generated by the arrival of filaments coming from the other pattern (so it indicates the contribution of the second pattern). (B) Results of simulations and experiments. (Left) Simulations with a distance of *d* = 7 μm between the two patterns for hypothesis H1 and H2. Filaments coming from opposite patterns are differently colored (white and yellow); filaments generated by the primer effect are depicted in green. (Right) Results of experiment for 3 different normalized distances between patterns *d/ d*_∞_: 0.3 (top), 0.6 (middle) and 1.2 (bottom). The actin organization is shown for two different times. (C) Comparison between the simulation and experiment outputs. Quantification of the experimental results, where the distance between the two patterns was varied from 5 to 120 μm (green points). Different symbols correspond to four different experiments. Quantification derived from 100 simulations for each case, as a function of *d*/*d*_∞_ where *d* was randomly chosen between 2 and 12 μm (lines). Under H1 (red line), *ρ*_*ext*_ does not depend on *d* and is near 0. On the contrary, under H2, (blue lines), *ρ*_*ext*_ is a decreasing function of *d* that is positive for *d < d*_∞_. Under H2, two different nucleation efficiencies were tested: a full efficiency (dashed blue line), and a 2.5% nucleation efficiency (percent of filaments reaching the opposing pattern able to trigger new nucleation; solid blue line). The vertical bar indicates the threshold where patterns were considered to be isolated.

We next simulated a pattern containing two parallel pWA (Arp2/3 activator) bars of width 3 μm to study the “primer effect”. This effect is based on the ability of a drifting actin filament to contact the nucleation area and trigger actin assembly (S2 and S3 Figs). The *in vitro* experiment was constructed such that a growing actin filament coming into contact with a virgin patterned region will nucleate new actin filament on its side [55]. However, when two or more patterned regions are in close proximity, a region of pWA may already be covered by an actin network (Fig 3A). We therefore have two possible scenarios for the fate of a filament reaching a pattern of pWA on which a branched network is already present (Fig 3A): (i) the filament gets entangled in this network and stop growing, (ii) the filament can nucleate new filaments as if the pattern was virgin (and get entangled or not). To find which scenario is correct, we compared simulations and *in vitro* experiments by systematically varying the distance between the two bars. As control condition, i.e. non-interaction between the two patterns, we selected cases where the distance *d* between adjacent bars is above (*d*_∞_ = 80 μm). In this case filaments from one bar do not reach the other bar.

To assess the contribution of a neighboring pattern on the overall actin network we computed:

$$\rho_{ext}\left(d\right)=\frac{I\left(\;d,\;t_{f}\right)}{I\left(\;d,\;t_{i}\right)}-\frac{I\left(\;d_{\infty },\;t_{f}\right)}{I\left(\;d_{\infty },\;t_{i}\right)}{\;}_{,}$$

which measures to what extent the outer (away from the neighboring bar) intensity is made different by the presence of the second bar (Fig 3A). For this we calculated the intensity of the network (≈density of filaments) close to the pattern (7 μm away from the pattern center; a pattern is 3 μm wide), at two different times *t*_*i*_ and *t*_*f*_ = *t*_*i*_ + 1*h* (Fig 3B). If the filaments cannot cross the pattern and get entangled, the variation of intensity at the outer sides of the pattern should be independent of the distance between the bars (Fig 3A) and thus similar to the control case (*ρ*_*ext*_(*d*) ≈ 0). On the other hand in case the filaments cross and/or nucleate on the other bar we expect the outer intensity to be higher than in the control case (*ρ*_*ext*_(*d*) > 0) and *ρ*_*ext*_(*d*) would be a decreasing function of *d*. Because the computational costs were too high, we simulated shorter and closer pattern bars than experimental ones. To allow comparison of the results, we looked at normalized distances (*d*/*d*_∞_).

We performed 100 simulations with the two different hypotheses, using a random distance *d* for each simulation (Fig 3B, and S5 Movie). We calculated *ρ*_*ext*_(*d*) for each simulation and analyzed its dependency on the normalized distance (Fig 3C) for the assumptions of entanglement (H_1_ solid red line) and nucleation (H_2_ dashed blue line). We then analyzed the results of four *in vitro* experiments (Fig 3B and 3C). The results are significantly positive for distances smaller than *d*_∞_ (with 95% confidence with Wilcoxon rank test for comparison of experimental results with null distribution), and decreasing with *d* (*p*-value < 10^–12^ with Spearman's rho test). We therefore found that the second hypothesis (H_2_, nucleation) best matches the experimental behavior. However, a simulated nucleation efficiency of 100% was too high according to the experimental data (Fig 3C). Thus, we decreased the efficiency down to 2,5% (Fig 3C, solid blue line). This predicted low value for “primer activation” is consistent with experimental quantification of the nucleation in presence of the Arp2/3 complex [48]. We therefore can conclude that *in vitro*, actin filaments may cross over neighboring bars, nucleate new filaments, and thus influence the network on the distal side of another patterned region.

## Discussion

To unveil how actin filaments can be organized into higher structures, a lot of efforts went into analyzing the role of actin-associated proteins [2,6,56]. While largely justified by the importance of protein effectors, this was also due to the lack of tools that allowed one to dissect how geometrical or physical parameters affect the overall actin architecture. However, recent technological developments such as microfluidics [57], micropatterning [1,58,59] or cytoskeleton growth into defined volumes [39] have highlighted some of the key features of biochemical-independent parameters in controlling the cytoskeleton architecture. Examples of applications include showing how chromatin shapes the mitotic spindles organization [60] or how actin nucleators of the formin family respond to mechanical stress [61]. Using a micro-printing technique, it was also possible to demonstrate that geometry is a key parameter in controlling the macroscopic architecture of actin filaments during assembly [1]. This made it possible to document the emergent behavior by which actin assembly organizes at higher scale, depending on the initial localization of the actin nucleation-promoting factors.

**Fig 4:**
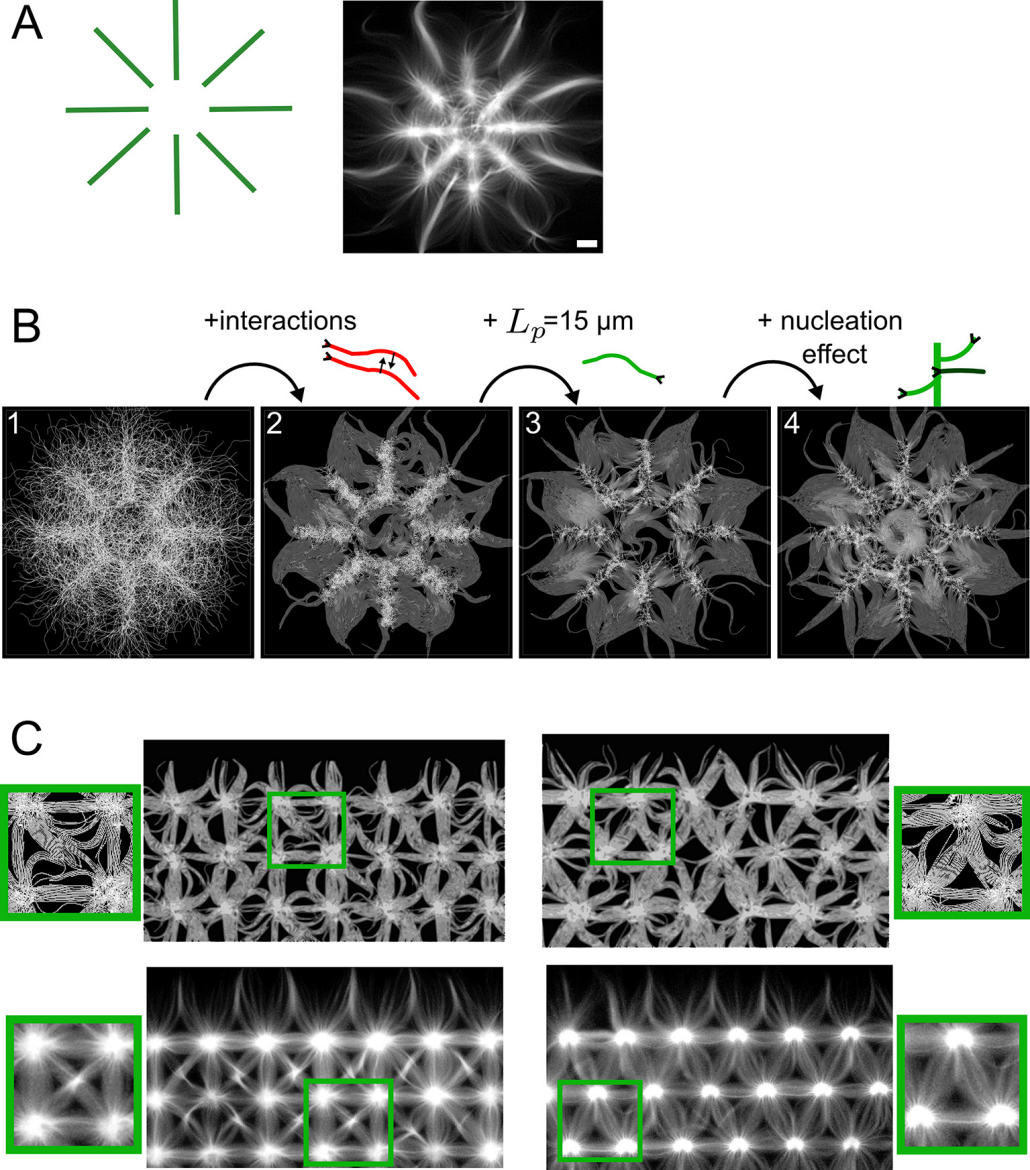
Contribution of studied parameters on actin organization. (A) Schematic of a micropattern, and the corresponding *in vitro* actin organization observed with TIRF microscopy. Scale bar is 10 μm. (B) The contribution of the key elements of the model on the final organization of fibers in the simulation. (B1) Fibers that are nucleated locally but do not interact with each other fail to extend away from the micropattern, (B2) Adding the steric interaction with the parameters as calibrated in Fig 1 allows filaments to co-align and extend away from the nucleation zone. (B3) Setting the persistence length to 15 μm (cf. Fig 2) makes them extend further. (B4) Adding the nucleation effect (cf. Fig 3, H2) finally leads to realistic overall densities (final time is 250 s). (C) Predictions of the actin filament organization for novel nucleation patterns. Simulations (Top) were done on a set of 8 points, distant by 6 μm, and positioned on a square (left) or triangular (right) lattice. The 8-point units were repeated 9 times using periodic boundaries. (Bottom) Experimental verification of actin organization for the geometries used.

Using Cytosim, we developed a model of a geometrically-constrained actin assembly, in which actin filaments/fibers are initiated from defined regions where branched nucleation occurs. This framework allowed us to study how the biophysical properties of actin filaments and their environment determine the organization of the final network. It is a tool with which one may study actin dynamics in more complicated systems. We were able to reproduce a diversity of actin organizations obtained from an initial geometry of nucleating promoting factor (Figs 1–3). We identified a combination of three essential components that determines the actin organization (Fig 4A and 4B and S6 Movie). The first one is the steric interaction between filaments, this is essential to obtain an aligned distribution of actin filaments growing away from the nucleating pattern (Figs 1 and 4B). In absence of steric interaction actin filaments have a tendency to buckle (Fig 4B1) preventing them from extending away from the nucleating region, and thus limits the contact between filaments nucleated from two different areas (Fig 4B1). These interactions are modulated *in vitro* by the chemical properties of the media. The second key component is the bending elasticity of actin bundles [62]. A persistence length of *L*_*p*_ ≈ 15 μm, close to the experimentally reported persistence length for single actin filaments [62], resulted in patterns with parallel and antiparallel actin organizations similar to the patterns observed *in vitro* (Figs 2 and 4B3). The model predicted the effect of regulatory proteins, such as ADF/cofilin [54] or crosslinkers [53] that modify the persistence length, on the observed patterns. Actin filaments decorated by ADF/cofilin are softer (*L*_*p*_ of 2 μm [54]) and will buckle under small force and we predicted that this would preclude the efficient formation of parallel or antiparallel organizations. On the contrary, a high persistence length (*L*_*p*_ = 1000 μm) induced for example by bundling of actin filaments in the presence of crosslinkers, limited the deformation of the growing polymers. In this case we predicted that the original geometry imposed by the micropattern is maintained, indicating that this is the main parameter that defines the final macroscopic organization during actin assembly. The effect of adding fascin in our system could be simulated by adapting only the fiber persistence length, without having to change the fiber steric interactions. This suggests that the electrostatic properties of the interaction could be negligible compared to the change in elastic property of the fibers, which arise from the capacity of fascin to generate stiff bundles [63]. For the native *L*_*p*_ ≈ 15 μm, the situation is more complex and the resulting actin macroscopic organization resulted from a combination of the boundary imposed by the geometry of the micropattern and the deformation induced on growing actin filament when contacting each other (Figs 2C, S4 and 4B3). Finally, a third component that is important in defining the overall actin macroscopic organization is the ability of growing actin fibers to contact the nucleating pattern and therefore trigger additional actin assembly following the ‘primer’ activation [55]. By doing so, the density of the actin organization will be modulated according to the efficiency of the ‘primer’ activation (Figs 3 and 4B4). The accessibility of the nucleating region is therefore a key parameter in controlling the overall actin organization. As demonstrated in Fig 4, the combination of these three components is sufficient to capture most of the actin organization observed *in vitro*, with the geometrically constrained actin nucleation assay.

To further test the power of our simulation tool, we predicted the overall collective behavior of actin initiated from more complex pattern geometry (Fig 4C). We simulated the actin filaments organization obtained from square and diamond dotted patterns (Fig 4C). We then constructed the same geometries experimentally (Fig 4C). In both simulations and experiments the network were organized similarly (See Fig 4C zoomed panel). This shows that our simulation framework is predictive. In its present form our simulations with Cytosim have a number of limitations. Firstly, the high computational demands (S2 Fig), lead us to simulate the model in 2D using a reduced number of effective filaments. This likely explains why the model could not fully reproduce the formation of very thick parallel actin bundles at the bisecting line between each ray of the 8 branched radial arrays (Fig 4). A 3D model, possibly reduced in size, but one in which single F-actin are simulated, could be used in the future to explore this further. However, even with these limitations, our current model is already useful to test a variety of geometrical configurations and predict the macroscopic organization of actin filaments. Cytosim was used here to mimic branched actin nucleation via Arp2/3, with minor modifications we could study other modalities such as formin dependent nucleation [64], the role of processive elongation induced by Vasp [65], or predict actin organization obtained by mixing several nucleation mechanisms. In the future we could investigate how myosin motors deform specific actin architectures [27] or study the movement of single myosin or myosin-driven cargo on established networks. Thus the present study sets the foundation of future research, where one will attempt to realistically reproduce *in silico* the emergent actin organization observed *in vitro*.

## Materials and Methods

### Simulation of filament growth from pattern

Actin filaments are simulated with Cytosim, following a Brownian dynamics approach [36]. Filaments may bend following linear elasticity, and are surrounded by an immobile viscous fluid; they grow and interact with each other (see Figs 1 and S2). Considering the low Reynolds number of a filament at this scale, inertia can be neglected and the mass of the objects is not a parameter that appears in the equations [37]. The parameters of the model were chosen to reflect the characteristics of *in vitro* actin filaments (see S1 Table). Notably, actin growth is force dependent, and the assembly speed is reduced exponentially by any antagonistic force [66] present at the barbed end:

$$v=\;v_{0}\;\text{exp}\left(\frac{f\cdot t}{f_{s}}\right),\;\text{if}\;f\cdot t\;<\;0\;\text{and}\;v=\;v_{0\;}\text{otherwise},$$

with *f*_*s*_ = 0.8 pN ([67]). ***f*** and ***t*** are respectively the force vector and the normalized tangent vector at the barbed end. Moreover, considering the extensive supply of monomers in the system, depletions effects were neglected, and it was not necessary to simulate actin monomers to correctly model the increase of “polymer mass” in the system.

To simulate the formation of patterns, three entities were used: nucleator objects, Arp2/3-like complexes, and binder objects (see S2 and S3 Fig). First, we placed randomly few nucleators on the pattern area, which will nucleate new ‘primer’ filaments. These nucleators can trigger the nucleation of one filament each, and have a fixed position so that they remain on the pattern. To simulate the branched network generated by activated Arp2/3 on the pattern, we placed Arp2/3-like objects in the pattern, which bind to any filament that is sufficiently close. Bound Arp2/3 object will then nucleate a new filament with an angle of 70° with respect to the other filament on which it is bound. These complexes are placed in a fixed position on the pattern, but will move with the filaments after binding, without inducing any constraint. The link between the mother and daughter filament is modeled as a Hookean spring, with a torque to constrain the angle between the branches. To add some friction on the pattern, we also placed on the pattern fixed binders that may bind and unbind to the filaments, thus restraining their motion. These binders are also modeled as Hookean springs between the fixed anchoring point, and the attachment location on the fiber.

S1 Table shows the important parameter values used in the simulations. The complete configuration file used to simulate a horizontal pattern (see Fig 1) is given in Supplementary Data.

### Simulation of steric interaction between filaments

The interaction between filaments is assumed to come from both depletion and electrostatic interactions. These are modeled in Cytosim as Hookean springs acting between the modeled segments, with an equilibrium distance *d*_0_ corresponding to the diameter of a fiber. Because of excessive computational costs (S2 Fig), we could not simulate all the filaments present in the system, and reduced their number by lowering the density by roughly a factor 10. For this, we used an effective diameter *d*_0_ = 100 *nm* (while the diameter of one actin filament is around 7 nm), and a maximum interaction range of *d*_*m*_ = 200 *nm* (while interactions between actin filaments mediated by electrostatic or depletion forces would be limited to tens of nm). Choosing *d*_0_ and *d*_*m*_, as well as the scale of the simulated system (length of the filaments, simulated time…) resulted from an empirical tradeoff between computational expenses and accuracy. With the chosen values, one filament usually interacts with its first and 2nd neighbors, and any modification of this aspect of the model can strongly affect the emergent organization. For example changing the value of *d*_*r*_ (defined from *d*_*m*_ = *d*_0_ + 2 *d*_*r*_, see S5A and S5B Fig) indicated a strong effect on filament bundling. Thus the adjustment between the filament radius, interaction range, and steric coefficient values is delicate, and the calibration procedure is essential to find appropriate values of those parameters. Note that with our parameter set, each simulated filament may represent ~10 neighboring actin filaments, and one should be careful while interpreting the results at a molecular scale.

### Simulation of different rigidities of actin filaments

We computed fibers of 3 different persistence lengths (2 μm, 15 μm, 1000 μm) to account for the effect of actin binding proteins. We calibrated the steric parameters for filaments having a native persistence length (15 μm), and kept these values fixed for the other persistence lengths. Indeed, we considered that the repulsive force, due to hard-core repulsion, should not be strongly affected by the addition of other proteins, so *K*_*push*_ could be kept constant for all rigidities. The electrostatic interaction term, *K*_*pull*_, could be affected by the addition of actin binding proteins if they do change the electronic charge of the filaments, or the depletion force. However, we do not have any measure of this potential effect to recalibrate this value, and keeping the same value was sufficient to explain the experimental observations in the presence of fascin. Moreover, we found that the range of steric parameters giving patterns similar to the one observed *in vitro* was minimally affected by the value of the persistence length (S6A and S6B Fig).

The increased persistence length due to fascin addition could have been modeled by true addition of crosslinker in our simulation, or by increasing the value of *K*_*pull*_. However, to be able to compare the behavior of fibers with different persistence lengths, we preferred to keep as many parameters as possible constant, and to vary instead the persistence length.

In all simulations we kept the same density of fibers. Therefore we do not explicitly model the decrease in fiber number that is expected from bundling. This choice was motivated by the computational costs of simulating large number of fibers. For native persistence lengths we simulated 300 to 1700 fibers, a 10 times decrease in case of bundling, i.e. 30 to 170 fibers, will not allow for comparison of the actin organizations. Instead we compared the behavior of single fibers against bundle of fibers for a same density of fibers/bundle of fibers.

We also tested how sensitive the simulations were to the chosen value of the filament persistence length, by varying it between 2 μm and 1000 μm (S7 Fig). We noticed that above a threshold, around 10 μm for the persistence length, the collective behavior of actin filaments was mostly similar with the same tendency of forming bundles (S7 Fig). Thus the choice of a precise value of 15 μm while actin persistence length is evaluated to be between 10 and 20 μm is acceptable.

### Simulation of entanglement and primer effect

To simulate the entanglement effect on the pattern and its effect on actin elongation (Fig 3), we defined two different fiber types in Cytosim. Their parameters are identical, but this allowed us to define an entity in the pattern that could bind at the barbed end of actin filaments coming only from the other pattern. When a filament has one or more of these entities bound close to its end, it will stop growing. For the second scenario (primer effect), we added the possibility for these entities to nucleate a new filament when bound. By mixing the two types of entities (capping one and nucleating one), we could control the nucleation efficiency.

## Supporting Information

S1 Fig *In vitro* organization on 3D view. (A) 3D reconstruction of filaments organization done with the software Chimera from confocal microscopy imaging. Color code illustrates the Z dimension. (B) Imaging in TIRF mode limits fluorescence to the filaments that are within ~200 nm of the coverglass surface plane. Scale bar: 10 μm
(TIF)

S2 Fig Description of the main processes that were simulated: patterning, nucleation, elongation, interaction. Cartoon representing the processes *in vitro* (left column), their implementation in Cytosim (middle column), and examples of simulations with a different implementation (right column). **Patterning:** The coverglass is covered by non-adhesive PLL-PEG polymer, and only the insolated area coated with pWA can activate the nucleation by Arp2/3 complex. Thus the patterned area is adhesive and creates friction on the filaments. We added binders (in blue) in the simulations to account for this friction (middle). We also added pre-activated complexes (orange) to initiate the nucleation. When the binders are removed, the region of high fiber density is not confined to the activated region (right). **Nucleation:** The contact between the inactive diffusing Arp2/3 complex, a pre-existing filament (primer) and the pWA allows the nucleation of a new filament by addition of diffusing monomer (left). Contact between pre-activated Arp2/3-like complex on the pattern and a filament generates nucleation of a new filament in Cytosim (middle). If the filaments are all nucleated without Arp2/3-like process (with fixed nucleators similar to those used as primers), we obtained a network that is very dense in the center, and all filaments are of similar lengths (right). **Elongation:** Under the *in vitro* conditions, with the concentration of 1–2 μM of actin, the elongation speed of filaments stayed approximately constant during the experiment (left). The growth is thus simulated with a constant rate (middle). If we added the constraint of limited amount of monomers, the filament growth slowed down during the simulation, and we obtained shorter filaments, but the global organization remained similar (right). **Interaction:** Filaments are attracting each other by electrostatic interactions due to the presence of counterions in solution and depletion force due to polymers (left). This is modeled by short range interactions calculated for each segment of a fiber in Cytosim (middle). If we remove this interaction, filaments remain disorganized (right). **Simulation**: Computation time needed to simulate a network of filaments for 150 s, as a function of the number of filaments in this network (middle). Final organization of filaments from a horizontal pattern bar with default parameters of our system (control, right).
(TIF)

S3 Fig Simulation of actin nucleation from pattern. Time-course of a simulation with actin filament nucleated from a rectangular area. Arp2/3-like entities are represented as small orange dots. Binders are represented as blue dots. At early times (t = 1 s), few nucleators entities randomly distributed generate short actin filaments (primers, shown in white). Upon contact with Arp2/3-like entities (t > = 10 s), these filaments are amplified by triggering the nucleation of new filaments; daughter filaments make a 70° angle with the mother filament. A zoom of a small part of the pattern is shown below. Scale bar is 1 μm.
(TIF)

S4 Fig Effect of the ratio between fiber length and persistence length on the collective organization. Proportion of parallel fibers as function of pattern angle *θ*, with a persistence length ***L***_***p***_ of 2 μm and 1000 μm, while the lengths of filaments is ***L*** = 7 μm. The ratio is defined as ***r* = *L***_***p***_ **/*L***. (B) Proportion of parallel filaments as a function of pattern angle *θ* for different fiber lengths and native persistence length. (C) Illustration of the collective organization for different ratios ***r*** (bottom).
(TIF)

S5 Fig Effect of steric parameters. (A) Effect of varying the steric range *d*_*r*_ between 10 and 100 nm. All other parameters are identical to Fig 1E. Scale bar is 4 μm. (B) Effect of varying the steric range *d*_*r*_, around its set value of 50 nm.
(TIF)

S6 Fig Effect of steric parameters on non-native persistence length of fibers. Variation of steric parameters for filaments with a persistence length of 1000 μm (A) or 2 μm (B). Three values were tested for *K*_*pull*_ (0.05, 0.5 and 5 pN/μm) and three values for *K*_*push*_ (0.75, 7.5 and 75 pN/μm), resulting in 9 combinations. The nucleating bar is 8 μm long and the image shows in grey levels the simulated actin density at 250 s (scale bar is 6 μm).
(TIF)

S7 Fig Sensitivity of actin organization on the fiber persistence length. Organization of actin fibers with 5 different persistence lengths: 2 μm, 5 μm, 10 μm, 15 μm and 1000 μm (from left to right). Pattern bar length is 8 μm and the image shows in grey levels the simulated actin density at 250 s.
(TIF)

S1 Movie Growth of actin filaments from pattern after calibration. Growth of filaments nucleated from a rectangle, with parameters chosen as discussed in Fig 1. Pattern bar length is 8 μm, 510 actin filaments are nucleated on the pattern. Simulation runs for 250 s.
(MP4)

S2 Movie Growth of actin filaments from a V-shape with persistence length of 15 μm. Filament persistence length is 15 μm; each rectangle is 8 μm long, nucleating 360 actin filaments. Simulations run for 250 s. Angle between pattern is 20° (left), 45° (middle) and 90° (right).
(MP4)

S3 Movie Growth of filaments from a V-shape with persistence length of 2 μm. Actin filament persistence length is 2 μm; each rectangle is 8 μm long, nucleating 360 actin filaments. Simulations run for 250 s. Angle between pattern is 20° (left), 45° (middle) and 90° (right).
(MP4)

S4 Movie Growth of actin filaments from a V-shape with persistence length of 1000 μm. Actin filament persistence length is 1000 μm; each rectangle is 8 μm long, nucleating 360 actin filaments. Simulations run for 250 s. Angle between pattern is 20° (left), 45° (middle) and 90° (right).
(MP4)

S5 Movie Growth of actin filaments from pattern under entanglement and nucleation assumptions. Growth of actin filaments from two parallel rectangles; each rectangle is 5 μm wide, nucleating 135 actin filaments. Simulations run for 500 s. The top panel was simulated under the entanglement assumption. The bottom panel was simulated under the nucleation assumption. Additional actin filaments whose nucleation is triggered when actin filaments reach the neighboring rectangles are shown in green.
(MP4)

S6 Movie Growth of actin filaments from a star-like shaped pattern. Growth of actin filaments from a star-like pattern based on the calibrated parameters of this study. The pattern is composed of 8 rectangles 8 μm in length, nucleating 230 or more actin filaments each. Simulation time is 250 s.
(MP4)

S1 Table Main parameters used in the simulation. (DOCX)
