# Supplementary figures and images for: Geometrical and Mechanical Properties Control Actin Filament Organization
Source: PLoS Comput Biol. 2015 May 27;11(5):e1004245. doi: 10.1371/journal.pcbi.1004245 (PMC4446331; doi:10.1371/journal.pcbi.1004245)

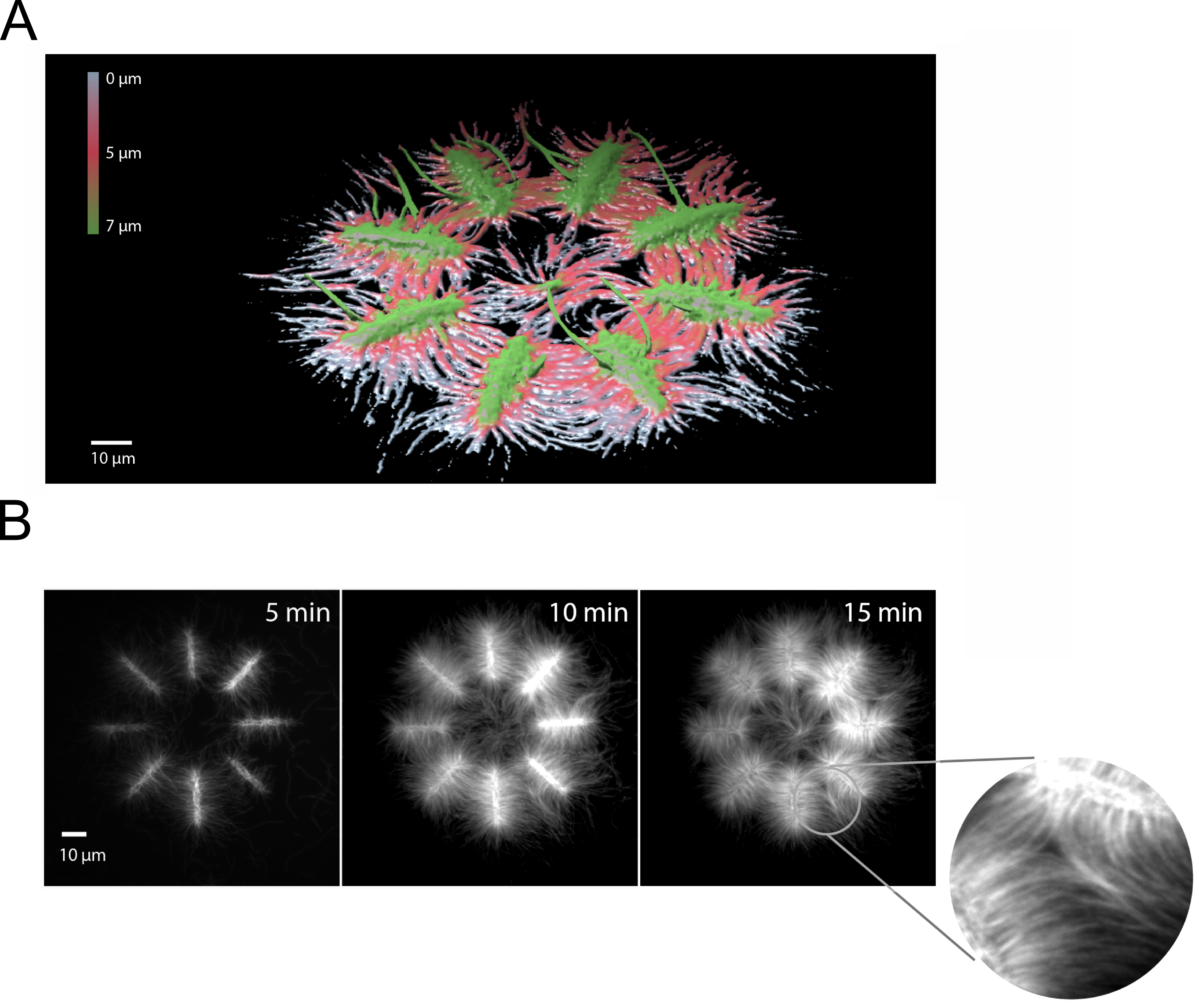

Supplement: S1 Fig — (A) 3D reconstruction of filaments organization done with the software Chimera from confocal microscopy imaging. Color code illustrates the Z dimension. (B) Imaging in TIRF mode limits fluorescence to the filaments that are within ~200 nm of the coverglass surface plane. Scale bar: 10 μm (TIF) [file pcbi.1004245.s001.tif]

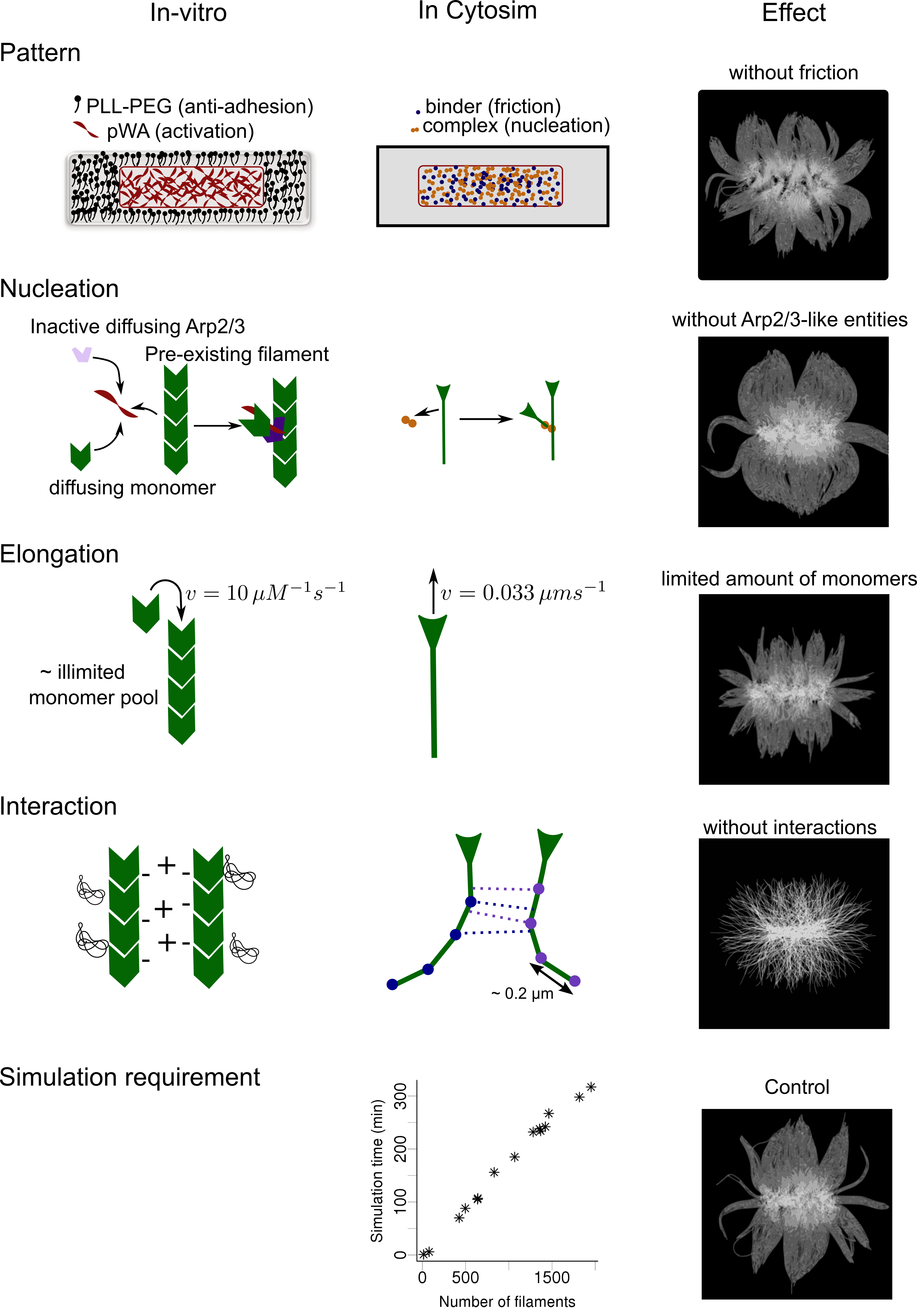

Supplement: S2 Fig — Cartoon representing the processes in vitro (left column), their implementation in Cytosim (middle column), and examples of simulations with a different implementation (right column). Patterning: The coverglass is covered by non-adhesive PLL-PEG polymer, and only the insolated area coated with pWA can activate the nucleation by Arp2/3 complex. Thus the patterned area is adhesive and creates friction on the filaments. We added binders (in blue) in the simulations to account for this friction (middle). We also added pre-activated complexes (orange) to initiate the nucleation. When the binders are removed, the region of high fiber density is not confined to the activated region (right). Nucleation: The contact between the inactive diffusing Arp2/3 complex, a pre-existing filament (primer) and the pWA allows the nucleation of a new filament by addition of diffusing monomer (left). Contact between pre-activated Arp2/3-like complex on the pattern and a filament generates nucleation of a new filament in Cytosim (middle). If the filaments are all nucleated without Arp2/3-like process (with fixed nucleators similar to those used as primers), we obtained a network that is very dense in the center, and all filaments are of similar lengths (right). Elongation: Under the in vitro conditions, with the concentration of 1–2 μM of actin, the elongation speed of filaments stayed approximately constant during the experiment (left). The growth is thus simulated with a constant rate (middle). If we added the constraint of limited amount of monomers, the filament growth slowed down during the simulation, and we obtained shorter filaments, but the global organization remained similar (right). Interaction: Filaments are attracting each other by electrostatic interactions due to the presence of counterions in solution and depletion force due to polymers (left). This is modeled by short range interactions calculated for each segment of a fiber in Cytosim (middle). If we remove this interacti [file pcbi.1004245.s002.tif]

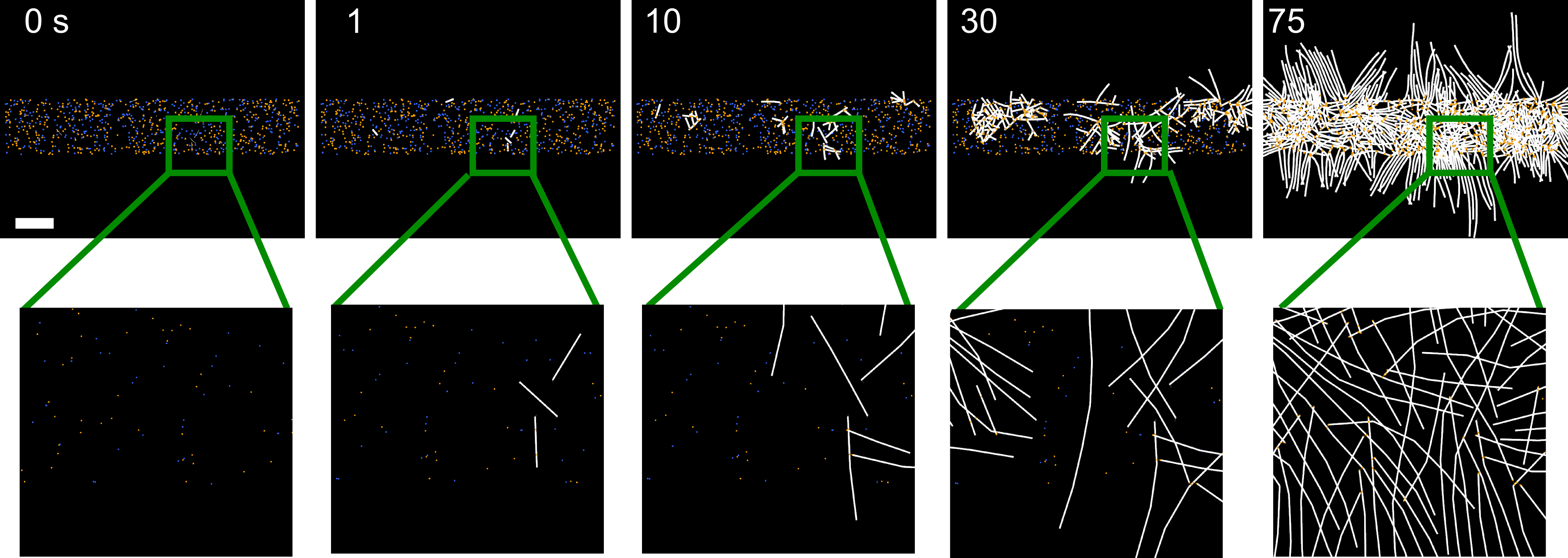

Supplement: S3 Fig — Time-course of a simulation with actin filament nucleated from a rectangular area. Arp2/3-like entities are represented as small orange dots. Binders are represented as blue dots. At early times (t = 1 s), few nucleators entities randomly distributed generate short actin filaments (primers, shown in white). Upon contact with Arp2/3-like entities (t > = 10 s), these filaments are amplified by triggering the nucleation of new filaments; daughter filaments make a 70° angle with the mother filament. A zoom of a small part of the pattern is shown below. Scale bar is 1 μm. (TIF) [file pcbi.1004245.s003.tif]

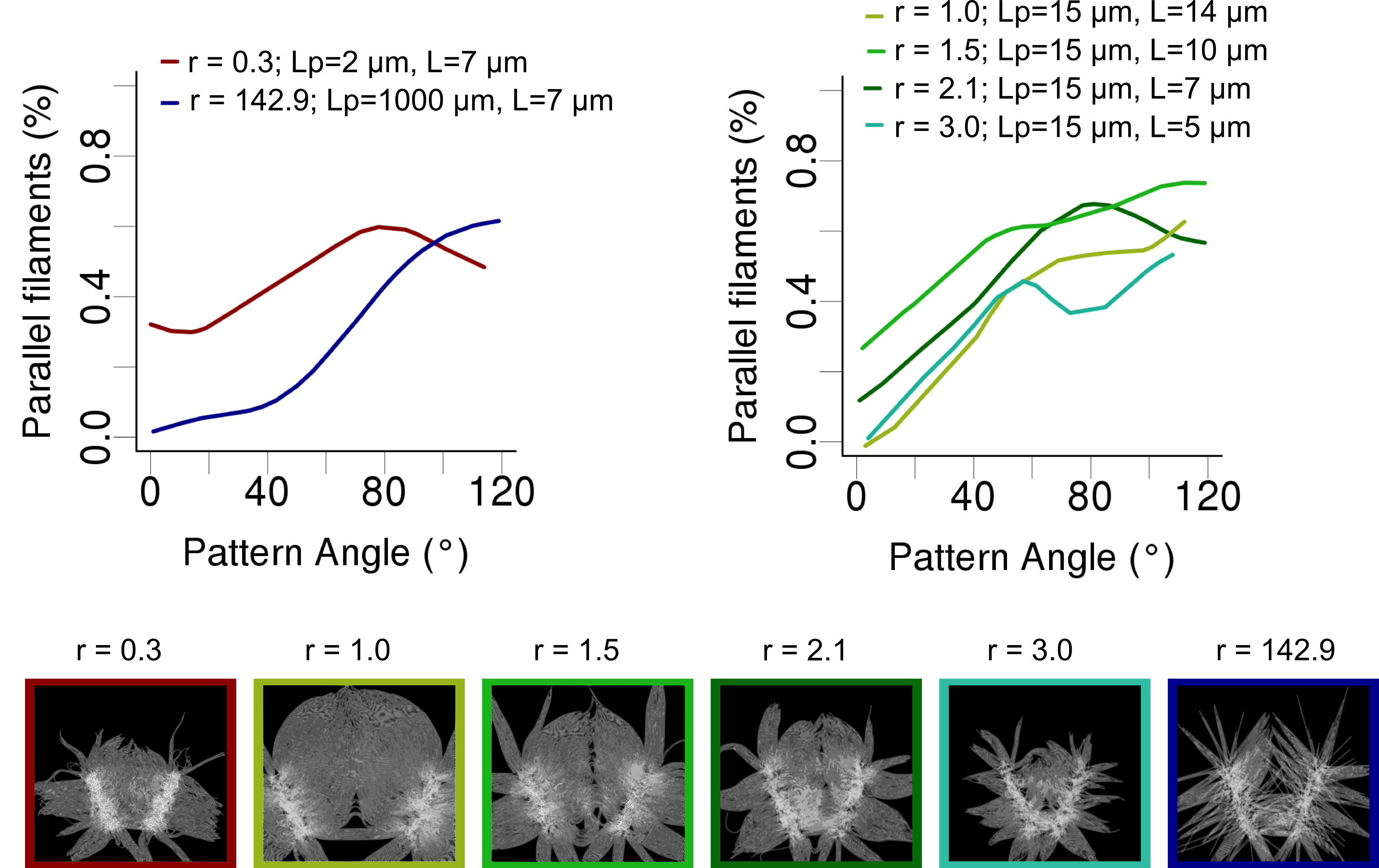

Supplement: S4 Fig — Proportion of parallel fibers as function of pattern angle θ, with a persistence length L p of 2 μm and 1000 μm, while the lengths of filaments is L = 7 μm. The ratio is defined as r = L p /L. (B) Proportion of parallel filaments as a function of pattern angle θ for different fiber lengths and native persistence length. (C) Illustration of the collective organization for different ratios r (bottom). (TIF) [file pcbi.1004245.s004.tif]

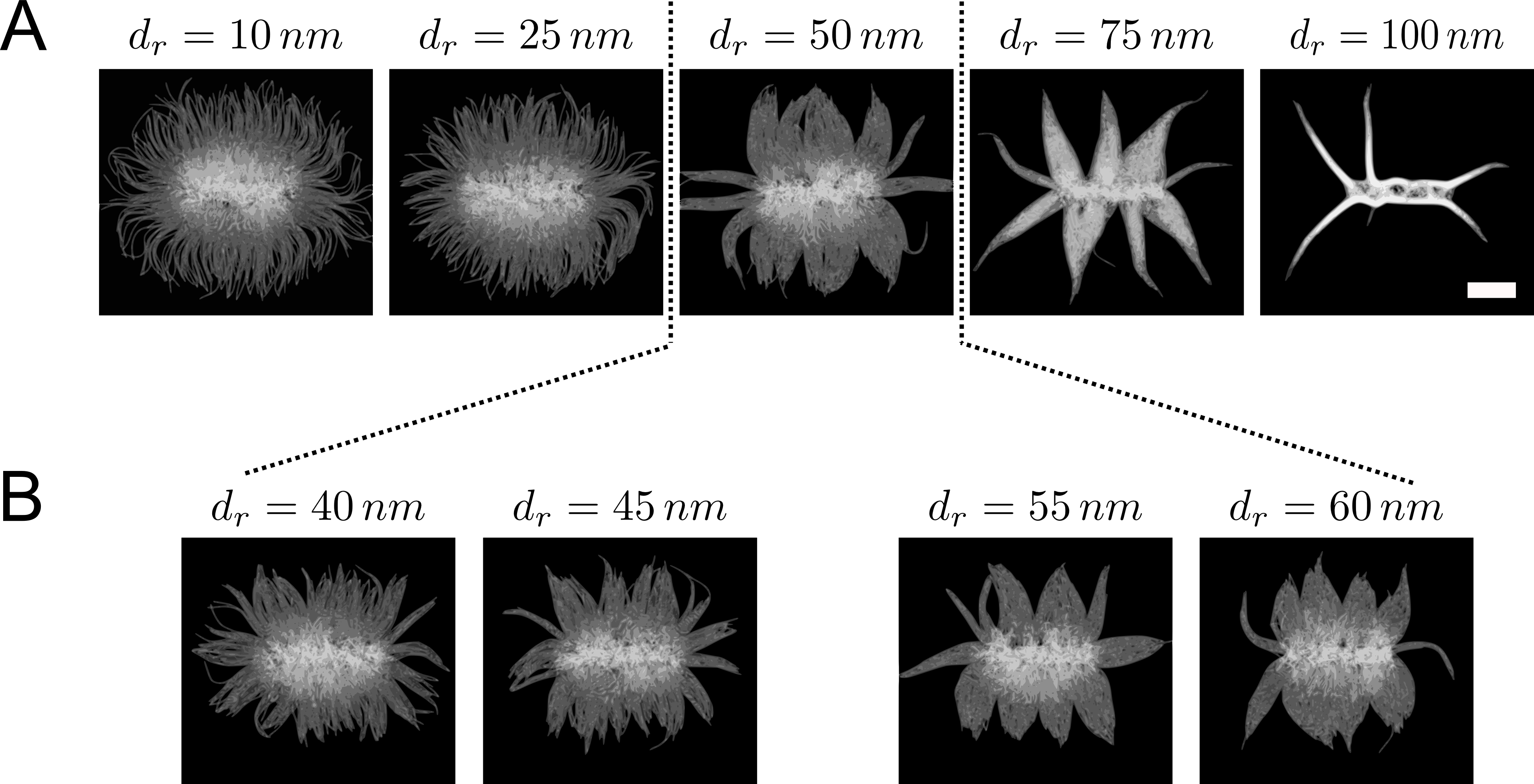

Supplement: S5 Fig — (A) Effect of varying the steric range d r between 10 and 100 nm. All other parameters are identical to Fig 1E. Scale bar is 4 μm. (B) Effect of varying the steric range d r, around its set value of 50 nm. (TIF) [file pcbi.1004245.s005.tif]

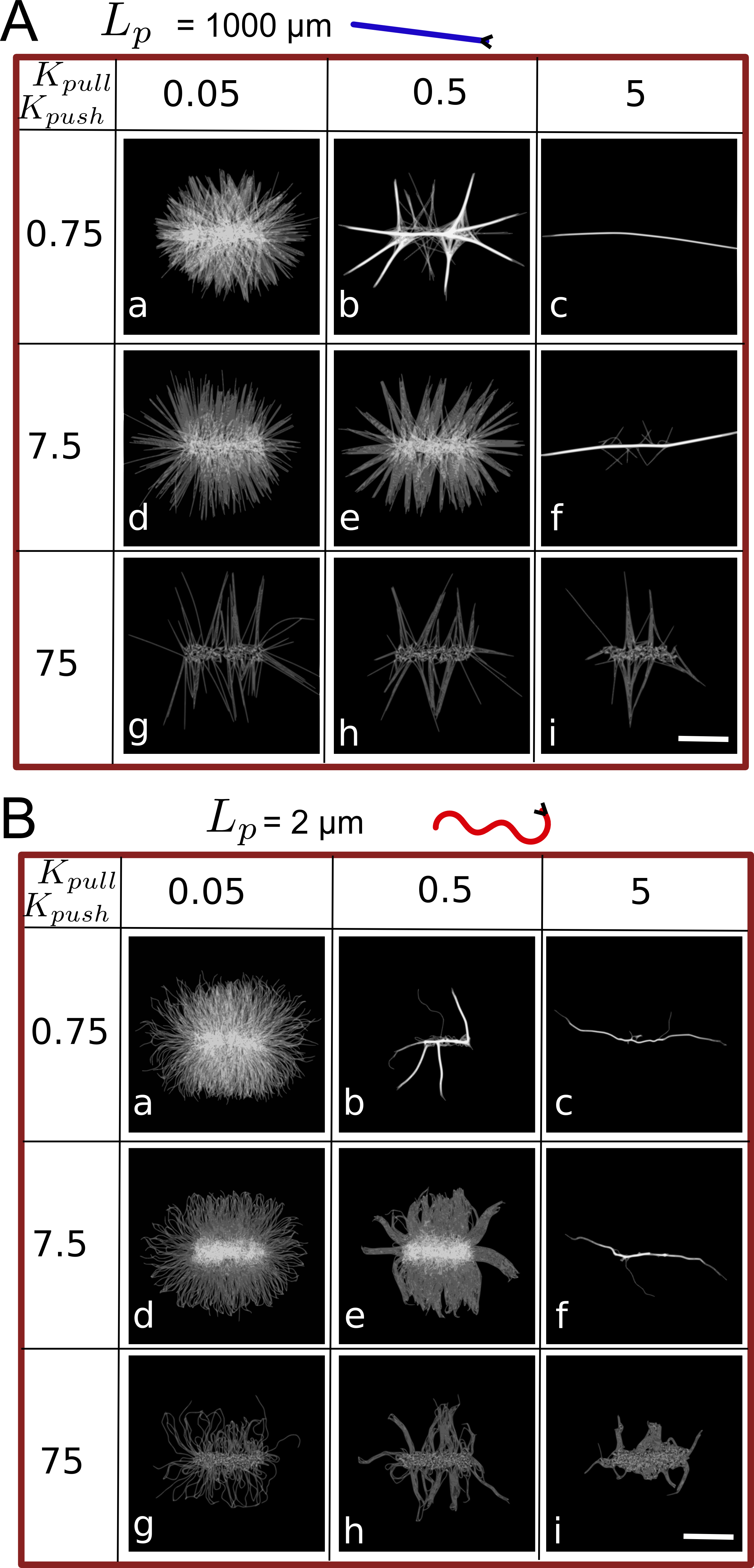

Supplement: S6 Fig — Variation of steric parameters for filaments with a persistence length of 1000 μm (A) or 2 μm (B). Three values were tested for K pull (0.05, 0.5 and 5 pN/μm) and three values for K push (0.75, 7.5 and 75 pN/μm), resulting in 9 combinations. The nucleating bar is 8 μm long and the image shows in grey levels the simulated actin density at 250 s (scale bar is 6 μm). (TIF) [file pcbi.1004245.s006.tif]

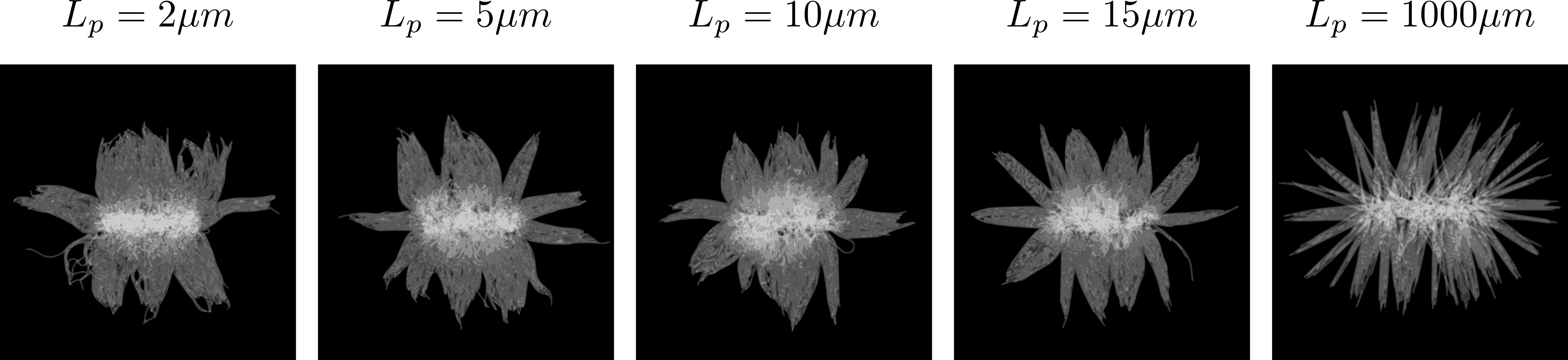

Supplement: S7 Fig — Organization of actin fibers with 5 different persistence lengths: 2 μm, 5 μm, 10 μm, 15 μm and 1000 μm (from left to right). Pattern bar length is 8 μm and the image shows in grey levels the simulated actin density at 250 s. (TIF) [file pcbi.1004245.s007.tif]
