# Supplementary material for: Geometrical and Mechanical Properties Control Actin Filament Organization
Source: PLoS Comput Biol. 2015 May 27;11(5):e1004245. doi: 10.1371/journal.pcbi.1004245 (PMC4446331; doi:10.1371/journal.pcbi.1004245)
Supplement: S1 Table — (DOCX) [file pcbi.1004245.s014.docx]

**S1 Table. Main parameters used in the simulation.**

| **Name** | **Value** | **Description** |
| --- | --- | --- |
| **Global** |  |  |
| Time step | 0.01 s | Computational parameter |
| Viscosity | 0.18 pN s/µm² | Viscosity of the solution containing methylcellulose |
| kT | 0.0042 pN µm | Thermal energy at 25°C |
|  |  |  |
| **Actin filaments** |  |  |
| Rigidity | 0.06 pN µm² | Flexural rigidity: Lp * kT, whith Lp, actin persistence length, chosen as 15 µm [62].  Varied in Figure 2 and Figure S6. |
| Segmentation | 0.2 µm | Computational parameter |
| Growing speed | 0.033 µm/s | Elongation speed under no force ($v_{0}$), for 1 µM of actin monomers [49]. |
| Growing force | 0.8 pN | Stall force ($f_{s}$) [67]. Growing velocity is slowed down by loading force on barbed end:$v= v_{0} exp(\frac{f}{f_{s}})$ |
| Steric diameter | 100 nm | Simulated fiber diameter $d_{0}$, discussed in Material and Methods |
| Steric maximal range | 200 nm | Maximal interacting distance, $d_{m}$, discussed in Material and Methods |
| Steric parameters |  | See Figure 1, S2 and Material and Methods |
|  |  |  |
| **Fixed nucleator** |  |  |
| Nucleation rate | 1 s^-1^ | Rate of nucleation, fast to simulate primers. Empirical |
| Unbinding rate | 0 $s^{-1}$ | No detachment |
| Stiffness | 30 pN/µm | Stiffness of the Hookean spring to attach the nucleator to its position. Empirical, low so that the initial position will not be a strong constraint |
| Number | 10 | Few primers |
|  |  |  |
| **Arp2/3 complex** |  |  |
| Nucleation rate | 0.5 $s^{-1}$ | Nucleation rate when bound to an existing filament |
| Binding rate | 1$s^{-1}$ | Fast binding rate for an efficient covering of the pattern area |
| Binding range | 0.02 µm | Can bind only to a close filament |
| Unbinding rate | 0 $s^{-1}$ | No Arp2/3 debranching |
| Equilibrium angle | 1.24 rad | Equilibrium angle between the two branches (70°, [48]) |
| Angular stiffness | 0.13 pN.µm/rad | Stiffness of the torque connecting the two branches [48] |
| Density (or number) | Between 300 and 1700 |  |
|  |  |  |
|  |  |  |
| **Binders** |  |  |
| Binding range | 0.02 µm | Maximal distance to which a binder can bind a filament. Empirical |
| Binding rate | 7 s^-1^ | Rate of binding of a binder to any filament that is within the binding range distance. Empirical |
| Stiffness | 100 pN/µm | Stiffness of the Hookean spring between the binder and the filament. Empirical |
| Unbinding rate | 0.01 s^-1^ | Rate of unbinding of the binders. Empirical |
| Stiffness | 100 pN/µm | Stiffness of the Hookean spring between the binder and the filament. Empirical |
| Number | 400 | Enough to create friction |
|  |  |  |
| **Simulation** |  |  |
| Number of filaments | Between 300 and 1700 | Range of number of filaments simulated (nucleated from Arp2/3 complex entities) |
| Total time simulated | Between 250 s and 500 s |  |
|  |  |  |
